# A machine learning method for estimating the probability of presence using presence‐background data

**DOI:** 10.1002/ece3.8998

**Published:** 2022-06-16

**Authors:** Yan Wang, Chathuri L. Samarasekara, Lewi Stone

**Affiliations:** ^1^ 5376 School of Science RMIT University Melbourne Victoria Australia

**Keywords:** constrained LK method, local certainty, local knowledge, presence‐background, prevalence, probability of presence, RSPF

## Abstract

Estimating the prevalence or the absolute probability of the presence of a species from presence‐background data has become a controversial topic in species distribution modelling. In this paper, we propose a new method by combining both statistics and machine learning algorithms that helps overcome some of the known existing problems. We have also revisited the popular but highly controversial Lele and Keim (LK) method by evaluating its performance and assessing the RSPF condition it relies on. Simulations show that the LK method with the RSPF assumptions would render fragile estimation/prediction of the desired probabilities. Rather, we propose the local knowledge condition, which relaxes the predetermined population prevalence condition that has so often been used in much of the existing literature. Simulations demonstrate the performance of the new method utilizing the local knowledge assumption to successfully estimate the probability of presence. The local knowledge extends the local certainty or the prototypical presence location assumption, and has significant implications for demonstrating the necessary condition for identifying absolute (rather than relative) probability of presence from presence background without absence data in species distribution modelling.

## Graphical abstract

We proposed a new concept of local knowledge that extends the local certainty or the prototypical presence location assumption, and has significant implications for demonstrating the necessary condition for identifying absolute (rather than relative) probability of presence without absence data in species distribution modelling.

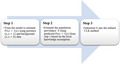

## 1 INTRODUCTION

Ecologists and environmental scientists make use of species distribution models (SDMs) to help understand how organisms selectively use their resources and the factors which shape their spatial distributions. SDMs have therefore become fundamental for many tasks in modern ecological modelling (Elith et al., 2006; Lele & Keim, 2006; Phillips & Elith, 2013). Considerable research has been carried out on species distribution modelling, as outlined in the extensive literature review of Guillera‐Arroita et al. (2015). Of the different types of spatial data used in SDMs, presence‐background (PB) data are plentifully available and easy to access. PB data consist of a list of “presences” or locations where individuals/species have been observed, but there is no information about locations of absences Gurutzeta. This latter characteristic makes the data difficult to work with. PB data are often available from museum, herbarium collections, and other historical database records. It now becomes increasingly available via citizen science projects and online repositories such as the Global Biodiversity Information Facility (GBIF: http://www.gbif.org). This paper will focus on the methodologies developed that relate to PB data, which have been used in approximately 50% of SDM papers, according to the survey in Guillera‐Arroita et al. (2015). When using PB data, the key objective is usually to accurately estimate the site‐specific probability of presence in a geographical area.

Numerous methods have been developed for modelling PB data with SDMs, including statistical regression models known in the literature as the LI (Lancaster & Imbens, 1996), LK (Lele & Keim, 2006), Expectation‐Maximization (EM) (Ward et al., 2009), Scaled Binomial Loss Model (SB) (Phillips & Elith, 2011), spatial point process models (PPMs) (Renner & Warton, 2013; Warton & Shepherd, 2010), machine learning (ML) methods such as MAXENT (Phillips et al., 2006), presence and background learning (PBL) algorithm (Li et al., 2011), boosted regression trees (Elith et al., 2008), and various other methods. It is commonly recognized that the actual (or absolute) probability of presence given environmental covariates, namely the resource selection probability function (RSPF), cannot be predicted from presence‐only or PB data, when there is no extra information or conditions available to make use of (Elith et al., 2008; Hastie & Fithian, 2013; Phillips & Elith, 2013; Wang & Stone, 2019; Ward et al., 2009). Without the extra information, all of these methods have been shown only to be useful in their ability to estimate the ratio between the probability of presence and the probability of prevalence, also known as the resource selection function (RSF) (Manly et al., 2002) or the “relative” probability of presence (Wang & Stone, 2019). In fact, the conditions required to estimate the true conditional probability of presence from PB data has become a highly controversial topic (Hastie & Fithian, 2013; Knape & Korner‐Nievergelt, 2015; Lele & Keim, 2006; Phillips & Elith, 2013; Solymos & Lele, 2015; Wang & Stone, 2019; Ward et al., 2009). LK claimed their method can successfully achieve this goal when the so‐called “RSPF conditions” are satisfied (Lele & Keim, 2006). (We will outline these conditions shortly.) A paper devoted to this question (Solymos & Lele, 2015) argues that the class of admissible models that satisfy RSPF conditions is very broad and does not excessively restrict the application of the LK method. However, this contradicts arguments in many other papers including the important paper of Hastie and Fithian (2013).

Wang and Stone (2019) recently revealed the close connection between many commonly used but seemingly disparate methods, such as the LI, LK, EM, SB, MAXENT, and PPMs. In particular, Wang and Stone (2019) proposed a new unified Constrained LK (CLK) method, which serves as a generalization of the better known existing approach. However, the CLK method requires information of the population prevalence as the input, which is difficult to obtain or estimate in practice. This renders the CLK method, along with other methods (such as the SB and EM methods) that also require prior information of the population prevalence, limited in their practical applications.

In this paper, we propose a “refined” CLK method that can be used to accurately estimate the population prevalence so that the probability of presence can be ultimately estimated. A key assumption of the method is that there exists “local knowledge” where habitats are maximally or partially suitable for a species. This means there must be some sites where we have knowledge about the resource selection probability of a species. The idea was inspired by the work of Elkan and Noto (2008) and Li et al. (2011). Elkan and Noto (2008) originally proposed the positive and unlabelled learning (PUL) algorithm to deal with the single‐training‐set sampling scenario in the field of ML. Li et al. (2011) further developed the presence and background learning (PBL) algorithm to estimate the actual probability of presence in modeling species’ distributions from PB data.

The performance of the method proposed here has been tested and compared with the popular LK method through extensive simulation studies, where a wide range of RSPFs resource selection probability functions were taken directly from Solymos and Lele (2015) to ensure that the RSPF conditions were satisfied according to their definitions in Solymos and Lele (2015). Our simulation studies reveal that the performance of the LK method is often poor even in situations where the RSPF conditions are satisfied, in contrast to the problematical claims of Solymos and Lele (2015). On the other hand, simulations demonstrate the ability excellent performance of the proposed method to estimate the absolute probability of presence when what we term “local knowledge” is satisfied. The experiments show that the local knowledge condition is not just helpful but is necessary for identifying probability of presence from PB data. Compared to the commonly used predetermined population prevalence (Phillips & Elith, 2011; Wang & Stone, 2019; Ward et al., 2009), the new method relaxes the required information and is thus less constrained.

## 2 MATERIALS AND METHODS

### 2.1 Description

When working with SDMs, we are interested in whether a species is present or absent at a particular site, conditional on environmental covariates (denoted by *x*). Here, the variable *y* = 1 represents the species’ presence while *y* = 0 represents its absence at a particular site. More specifically, a key goal is to estimate the conditional probability of Pr(y=1|x), namely the absolute probability of presence at a site, based on the covariate *x* measured at that site. The overall population prevalence will be denoted as π=Pr(y=1), that is, fraction of sites in the study area in which the species is present. A common practice in statistical modeling is to assume a parametric structure for Pr(y=1|x), for example, the logit function

(1) $$\text{Pr}\;\left(y=1|x;\beta \right)=\frac{1}{1+\exp\left[‐\eta \left(x;\beta \right)\right]},$$

where η(x;β) can be a linear or nonlinear function of *x*, and *β* are parameters that need to be estimated.

The form of the parametric function is critically important for some statistical methods in SDM such as the LK method (Lele & Keim, 2006), and can determine the success of the method. In contrast, our proposed model is less reliant on the explicit form of the parametric function. Examples will be provided in section 2.6 to show the robustness of our proposed method.

Presence‐background data contain two independent random samples. The first sample *P* of size *n*_1_ is a random sample drawn from the presence points according to a selection/labelling mechanism (i.e., being labelled in the language of ML). The second sample *B* of size *n*_0_ is drawn independently and identically from all locations in the study area with only the covariates but no information about presence or absence at each location. We use *s* to represent the sampling stratum, with *s* = 1 for the observed/labelled samples appearing in *P* and *s* = 0 for “unlabelled” samples in *B*. Pr(s=1) gives the probability of a species being observed or the probability of belonging to the presence samples. The class of *y* is not observed, but its information can be derived from the value of *s*. If the sample is labelled *s* = 1, then it is a “presence” with *y* = 1; that is Pr(y=1|s=1)=1. But, if s=0 we do not know whether y=1 or y=0. An important assumption underlying the labelling mechanism is that each labelled sample is chosen completely at random with the probability Pr(s=1|y=1). The “selected completely at random” (SCAR) assumption is stated formally as Pr(s=1|y=1,x)=Pr(s=1|y=1) (Bekker & Davis, 2020; Elkan & Noto, 2008). This assumption implies that no sampling bias exists for the PB data, or with sampling bias but the sampling effort and presence of species are conditionally independent given *x*. The latter assumption holds in many cases for PB data (Phillips et al., 2009).

This type of sampling mechanism was defined as the case‐control with contaminated controls in Lancaster and Imbens (1996) in econometrics studies and use‐availability sampling in habitat‐selection studies (Keating & Cherry, 2004). In the context of ML, this problem belongs to a special case of classification, that is, PBL or case‐control scenario of the PUL (Bekker & Davis, 2020; Elkan & Noto, 2008; Li et al., 2011).

### 2.2 Recap of the LK and CLK methods

The LK method defines the target log likelihood of the presence samples as (Lele & Keim, 2006);

$$\sum_{x\in P}\ln\text{Pr}\;\left(x|y=1;\beta \right).$$

By applying Bayes rule it becomes;

$$\sum_{x\in P}\ln\frac{\text{Pr}\;\left(y=1|x;\beta \right)\text{Pr}\;\left(x\right)}{\text{Pr}\;\left(y=1\right)}.$$

Dropping the terms that do not depend on the β, we obtain;

(2) $$\sum_{x\in P}\ln\frac{\text{Pr}\;(y=1|x;\beta )}{\text{Pr}\;\left(y=1\right)}.$$

Lele and Keim (2006) used the background data *B* to estimate the denominator term π=Pr(y=1) of population prevalence empirically, and obtained the following log‐likelihood;

(3) $$L=\sum_{x\in P}\ln\frac{\text{Pr}\;(y=1|x;\beta )}{\frac{1}{n_{0}}\sum_{x\in B}\text{Pr}\;(y=1|x;\beta )}.$$

In Equation (3), the species' prevalence has been approximated by the average probability over the background samples. Standard optimization techniques are used to find the coefficients ***β*** that maximizes Equation 3 (Lele & Keim, 2006).

Lele and Keim (2006) and Solymos and Lele (2015) argued that the LK method is valid in estimating the absolute probability of presence, if the so‐called RSPF conditions hold. When stated simply, the RSPF conditions include (1) the true (actual) function of logPr(y=1|x) should be nonlinear, and (2) not all covariates can be categorical. Readers are referred to Solymos and Lele (2015) for a complete discussion of these conditions. The logit function given in Equation (1) is one of the parametric functions that meet the RSPF conditions. (The complementary log‐log (cloglog) function is another example that meets the conditions.) When these conditions are satisfied, it is claimed that the LK method can estimate the absolute probability of presence of a species without any other extra information needed (Lele & Keim, 2006; Solymos & Lele, 2015). Despite this, Hastie and Fithian (2013) have given an in‐depth analysis showing why the LK method is incapable of estimating the correct parameter values when data arise via models that are nearly linear on the logit scale.

Existing literature makes it clear that information of population prevalence π is required in advance to estimate the absolute probability of presence at each site (Elith et al., 2008; Hastie & Fithian, 2013; Phillips & Elith, 2013; Wang & Stone, 2019; Ward et al., 2009). In Wang and Stone (2019), we proposed the Constrained LK (CLK) method, which also requires the population prevalence as the prior information. In detail, the CLK method maximizes the following (LK type of) likelihood function,

(4) $$L_{1}\left(\beta \right)=\sum_{x\in P}\frac{\text{Pr}\;(y=1|x,\beta )}{\frac{1}{n_{0}}\sum_{j=1}^{n_{0}}\text{Pr}\;(y=1|x_{j},\beta )},$$

with the constraint, i.e., $\frac{1}{n_{0}}\sum_{j=1}^{n_{0}}\;\text{Pr}\;\left(y=1|x_{j},\beta \right)=\pi_{0}$, where $\pi_{0}$ is a predetermined population prevalence and $n_{0}$ is the number of background samples. Note that this is different from just maximizing the function of Equation (3) in the LK method, as the constraint reduces the effective parameter space over which the maximization is performed. It was also illustrated in Wang and Stone (2019) that the CLK method is capable of estimating the true probability of presence, the same as the EM (Ward et al., 2009), SB (Phillips & Elith, 2011) and SC (Steinberg & Cardell, 1992) methods, when the population prevalence is known. However, the population prevalence, π, is difficult to obtain in practice.

### 2.3 Estimating prevalence

**FIGURE 1:**
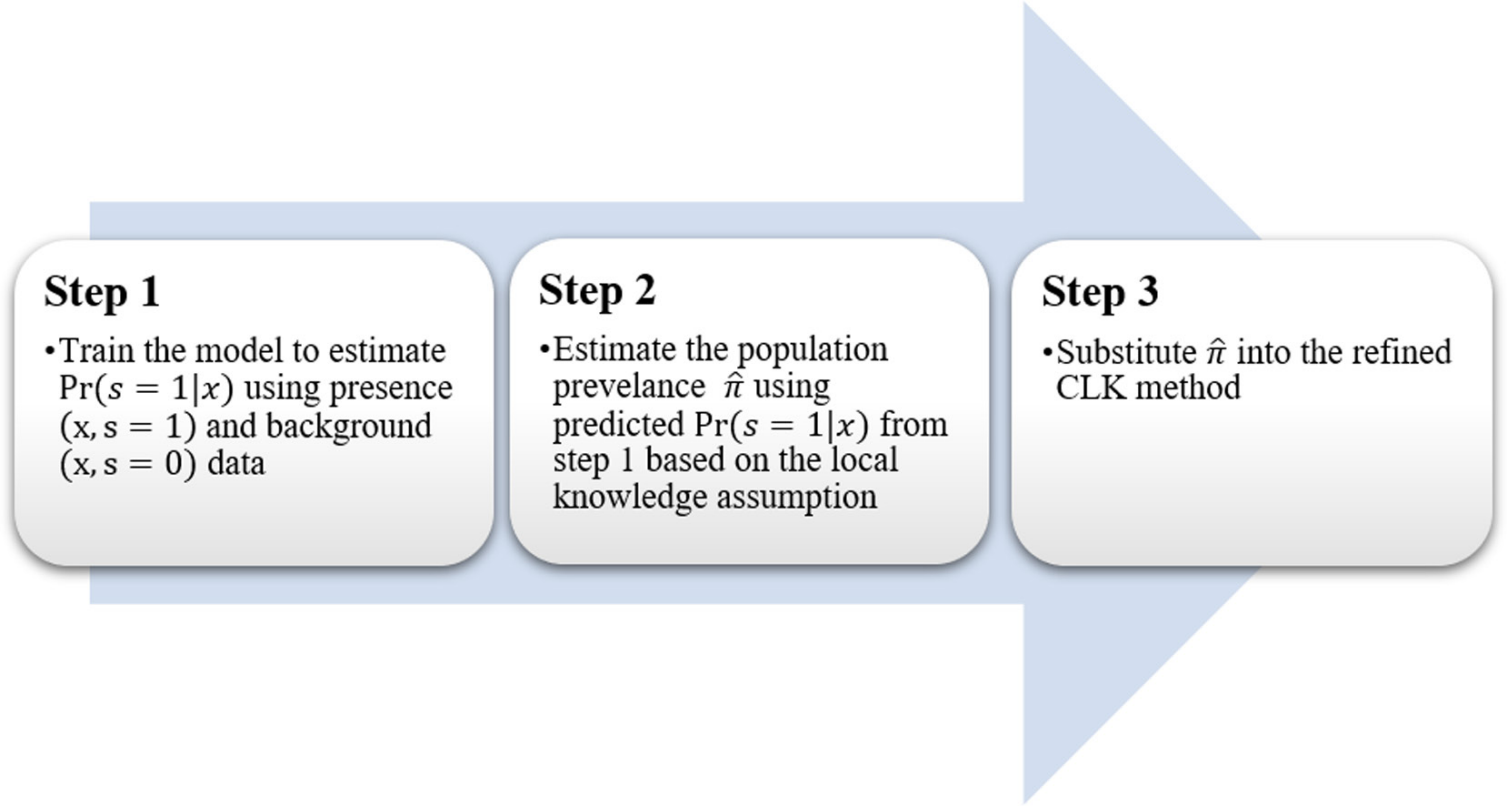
Steps for the refined CLK method to estimate the probability of presence from presence‐background data

Although it is difficult to obtain an accurate predetermined prevalence estimate of π in practice, we find the concept of “prototypical presence locations” (PPL) proposed by Li et al. (2011) often yields accurate estimates of π. The concept was inspired by the PUL algorithmpresence and background learning (PBL) (Elkan & Noto, 2008), and was further developed by Li et al. (2011) in the ecological context of modelling PB data (PBL algorithm) species distributions. In this section, we propose an estimate for the population prevalence π, which can be plugged into the CLK method or other methods that require a predetermined prevalence to ultimately estimate the absolute probability of presence. The steps of the new refined CLK method are summarised in Figure 1.

As a first step, we derive the relation between the probability of a species being observed, that is, Pr(s=1|x), and absolute probability of presence Pr(y=1|x) as follows (see derivation in Appendix A),

$$\text{Pr}\;\left(s=1|x\right)=\frac{1}{1+\frac{n_{0}}{p_{1}}\cdot \frac{\pi }{\text{Pr}\;(y=1|x)}},$$

where $p_{1}$ is the number of observed presences and $n_{0}$ is the number of background data. If we use $p_{2}$ to denote the number of true presences in $n_{0}$ background data, and *c* is defined as $c=\frac{p_{1}}{p_{1}+p_{2}}$, we can also obtain

(5) $$\text{Pr}\;\left(s=1|x\right)=\frac{1}{1+\frac{1‐c}{c}\cdot \frac{1}{\text{Pr}\;(y=1|x)}}.$$

Equation (5) shows the relation between the target probability of presence Pr(y=1|x) and the conditional probability of the observed presence Pr(s=1|x) under the case‐control sampling scheme. This key relationship was also derived by Phillips et al. (2009) and Li et al. (2011). The derivation in Phillips et al. (2009) led to the introduction of the scaled binomial (SB) method (Elith et al., 2011), which assumes the population prevalence π to be known.

Elkan and Noto (2008) and Li et al. (2011) proposed ML approach to estimate c, and a central assumption was employed in their approaches. Although not specifically defined in their work, Elkan and Noto (2008) assume that Pr(y=1|x) must equal to 1 at certain x value. This property was defined later by Bekker and Davis (2018) as the “local certainty” (LC) or the “positive subdomain/anchor set” assumption (Bekker & Davis, 2020). Similarly, Li et al. (2011) introduced the concept of PPL at which the habitat is maximally suitable for the given species to survive. In the statistical language, the conditional probability of presence at these PPL is one, that is, Pr(y=1|x)=1. In our framework, a similar assumption will be used to obtain an estimate for π.

At PPL, that is, when Pr(y=1|x)=1, it can be shown from Equation (5) that;

(6) Pr(s=1|x)=c.

In another word, Equation (6) shows that we can estimate c through the predicted values of Pr(s=1|x) at the PPL. For this, we first use ML methods to predict Pr(s=1|x) by training a binary classification model using the presence (x,s=1) and background (x,s=0) data. Popular classification methods, such as logistic regression, k‐nearest neighbours (KNN), support vector machines and neural networks, can be used to model Pr(s=1|x). In this paper, a neural network was used as the classifier which has been shown to perform very well.

One or more geographical locations should exist as the PPL but proper identification may be hindered by noise in the predicted values of Pr(s=1|x). To reduce the effect of noise, Elkan and Noto (2008) proposed three methods for estimating the sampling probability c, that is, the average values of Pr(s=1|x) at the PPL, the maximum values of Pr(s=1|x) at the PPL, and the ratio between the sum of Pr(s=1|x) at the PPL and the sum of Pr(s=1|x) over the background. Using the average as an example, *c* can be estimated as follows (Elkan & Noto, 2008; Li et al., 2011).

(7) $$\hat{c}=\frac{1}{n}\sum_{x\in \text{PPL}}\text{Pr}\;(s=1|x).$$

That is, c will be evaluated as the mean of predicted probabilities of Pr(s=1|x) for x that belongs to the PPL. According to Equation 5, the probability of Pr(y=1|x) is an increasing function of Pr(s=1|x), so we use the locations where Pr(s=1|x) are maximal as the PPL. We rank the predicted Pr(s=1|x) values for all presence and background points, and those locations where predicted Pr(s=1|x) are high are used as the PPL (Li et al., 2011). In our simulation study, we used the top 10th percentile, which is tested sufficient and appropriate for our study.

From Equation (6), we can easily show the relation between π and c as follows;

(8) $$\pi =\frac{p_{1}}{n_{0}}\cdot \frac{1‐c}{c},$$

π can thus be estimated as

(9) $$\hat{\pi }=\frac{p_{1}}{n_{0}}\cdot \frac{1‐\hat{c}}{\hat{c}}.$$

We then substituted $\hat{\pi }$ into Equation (4) as the constraint of the CLK likelihood function to ultimately estimate the unknown parametric function of Pr(y=1|x,β).

In our proposed method, the population prevalence is estimated from the available PB data, provided the LC condition is satisfied. This contrasts with previous studies in which a predetermined value of prevalence (although often unavailable) is required (Phillips & Elith, 2011; Wang & Stone, 2019; Ward et al., 2009). The LC or the PPL requires the maximal probability to be one at some sites. This might appear to be a strong assumption; however, we suggest that this assumption is not implausible both theoretically and practically.

### 2.4 Justification of LC/PPL Assumptions

In the relevant ML literature such as Elkan and Noto (2008) and Li et al. (2011), it is typically assumed that the LC or the PPL condition holds for general classification problems. However, these critical assumptions were not made explicit in the literature. In this section, we will explain how the PPL or the LC is not implausible in the context of species niche modelling. To understand the concept clearly, consider the classification problem when working with true presence and absence data. Suppose for example, a logistic function for Pr(y=1|x) (such as in Equation (1)) is the underlying probability functionresource selection probability function that drives the distribution of presence and absence observations. When we simulate the presence‐absence data from Pr(y=1|x), we first generate a random value r on the interval of [0, 1] for each site. A site is assigned y=1 (presence) if r≤Pr(y=1|x), and y=0 (absence) otherwise. Those sites with probabilities less than one could be randomly allocated as either presence or absence (or just absence) depending on the random value of r. The presences and absences generated at these sites would thus be “inseparable.” In another word, in order for (observed) presences distinguishable from absences (unobserved in PB data), some sites must have Pr(y=1|x)=1 (i.e., the PPL) so that a unique region in feature space is only occupied by the presence samples.

**FIGURE 2:**
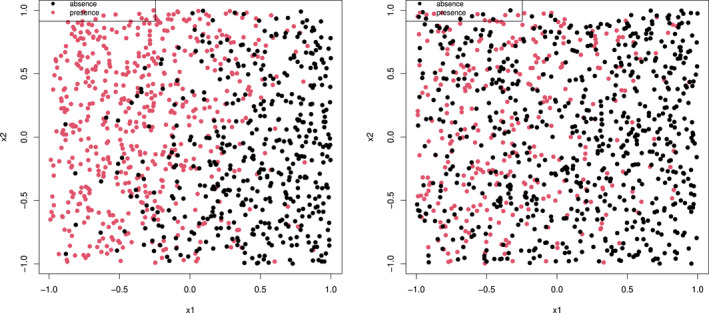
The presence (red dots) and absence (black dots) on the left were generated from a logistic probability function $\mathrm{Pr}\;\left(y=1|x\right)=\frac{1}{1+\exp(‐0.606+3.64x_{1}‐1.26x_{2})}$, which satisfies the LC condition, that is, Pr(y=1|x)=1 at some points. The presence and absences points in the right figure were generated from a scaled logistic probability function $\mathrm{Pr}\;\left(y=1|x\right)=\frac{0.5}{1+\exp(‐0.606+3.64x_{1}‐1.26x_{2})}$, where the probability of one is not reached in the feature space of $\left(x_{1},x_{2}\right)$. The presence (red) and absences (black) points are more separable in the left figure, particularly in the top left area; whereas there is no clear region in the right figure where presence and absence points are clearly separable

Figure 2 shows two presence‐absence datasets driven by two different probability functions. The first dataset was generated from a logistic probability distribution function that satisfies the LC condition (see left figure of Figure 2), whereas the second data points were generated from a scaled logistic function where the maximal probability does not reach one (see right figure of Figure 2). It can see from the first figure that there exists some PPL where the presence (red) points are clearly separated from the absence (black) points, particularly in the top left quadrant. In contrast, in the second dataset, there appears no region where presences are clearly separated from absences; presence and absence points nearly overlap in the feature space of $\left(x_{1},x_{2}\right)$.

Machine learning focuses more on classes that are strongly separable. Therefore, to classify presence and absence from the PB data, LC or PPL is a key assumption underlying the case‐control sampling scenario in PBL. Without extra information, the presence and absence points in the second data set would be hard to separate, regardless of the method used.

### 2.5 Local Knowledge (LKN) condition

In statistical studies, however, we notice that the LC or PPL condition is sometimes not satisfied, i.e., Pr(y=1|x) does not reach the maximal value of one. The scaled logistic example used in Hastie and Fithian (2013) to demonstrate the failure of the LK method and our second example in Figure 2 are such examples. When the presence and absence may not be distinguishable, other assumption or condition must be required to identify probability of presence from PB data. However, this condition should not be based on unfounded assumption, such as the RSPF conditions (Solymos & Lele, 2015). Rather in this section, we propose a local knowledge (LKN) condition that is one of such assumptions or conditions to handle these “difficult” problems. Compared to the commonly recognised population prevalence, which requires the probabilities of presence over the whole landscape, the LKN condition relaxes the information required and is thus less constrained.

A simple derivation from Equation (5) shows that;

(10) $$\pi =\frac{p_{1}}{n_{0}}\cdot \frac{1‐\text{Pr}\;(s=1|x)}{\text{Pr}\;(s=1|x)}\text{Pr}\;\left(y=1|x\right).$$

If we have the knowledge, for example, about the maximal probability of presence of the species (which is not necessarily one), we can rank the predicted values of Pr(s=1|x) for all observed presences and background points. Those locations with the highest Pr(s=1|x) will be used as the locations with LKN. Using a maximal probability of 0.7 as an example, we can show from Equation (10) that

(11) $$\hat{\pi }=\frac{0.7p_{1}}{n_{0}}\left[\frac{1‐\text{Pr}\;(s=1|x_{L})}{\text{Pr}\;(s=1|x_{L})}\right],$$

where L are those locations with LKN, for example, the maximal probabilities of Pr(y=1|x) in this case. LKN does not necessarily refer to the information of the maximal probability, and it can also include any nominated probability of presence at a given site or over a particular landscape. In general, if LKN is available so that there are some locations where the probabilities of Pr(y=1|x) are known, it should be possible to make use of the trained value of Pr(s=1|x) at these locations to estimate π from Equation (10) and to finally estimate our objective function of Pr(y=1|x). In the following simulation section, the scaled logistic example explored in Hastie and Fithian (2013) will be revisited to show how the LKN assumption can be used to estimate the absolute probability of presence. We also test the LKN on some probability functions extracted from Solymos and Lele (2015) that satisfy the RSPF conditions.

The LKN condition was built under the same framework and extends the LC condition. The PPL assumption given by Elkan and Noto (2008) and Li et al. (2011) is just one particular LKN subclass, in this case, where Pr(y=1|x)=1 at these locations. In practice, LKN could be gained through experts’ knowledge or pilot studies.

At the end of this section, we summarize the steps (as given in Figure 1) of the new refined CLK method to estimate the conditional probability of presence Pr(y=1|x,β) as follows:

Step 1: Train the model to estimate Pr(s=1|x), using all presence (x,s=1) and background (x,s=0) data points. A commonly used ML binary classifier, such as the neural network or support vector machine, can be used for modelling Pr(s=1|x);
Step 2: Estimate the population prevalence $\hat{\pi }$ using Equation 10 based on the predicted probabilities of Pr(s=1|x) at the PPL or locations with LKN;
Step 3: Maximize the log‐likelihood function of Equation (4) with respect to β, with π substituted by $\hat{\pi }$ from step 2 and obtain the estimated probability of presence Pr(y=1|x;β).

### 2.6 Simulation study

In this section, simulations are carried out first to assess the fragility of the LK method and its RSPF conditions proposed by Lele and Keim (2006) and Solymos and Lele (2015); and second to demonstrate the performance of the proposed concept of LKN, in conjunction with the CLK method, to estimate the probability of presence.

**FIGURE 3:**
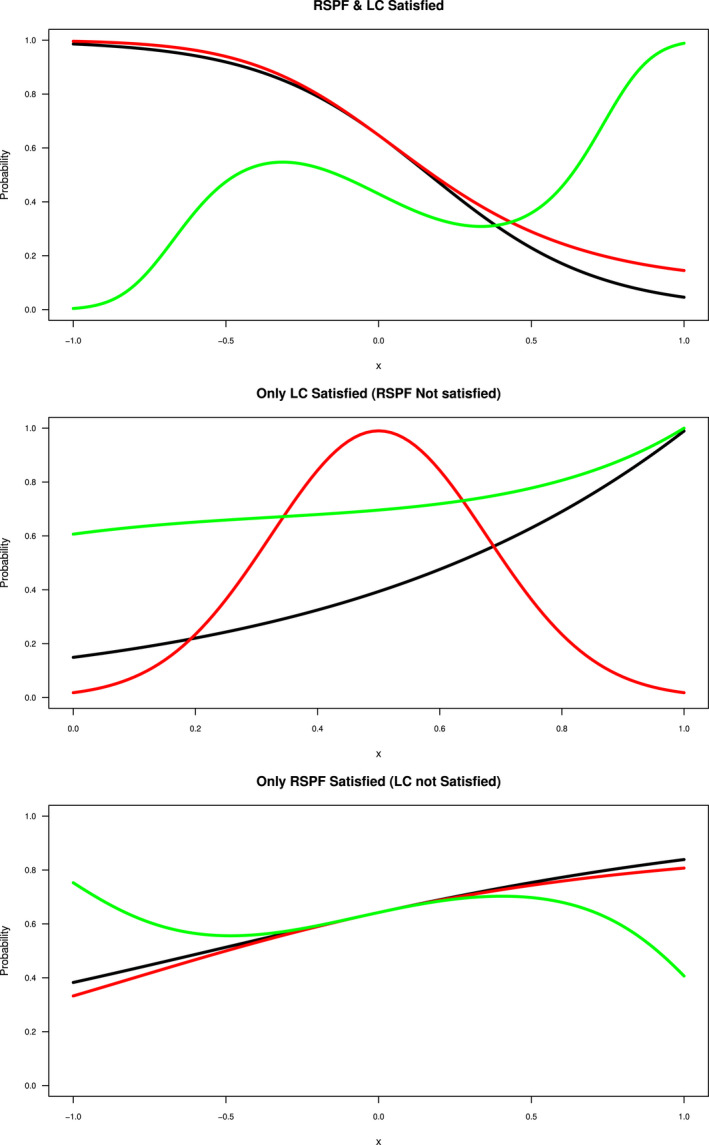
The selected simulated species distributions of the three categories of functions. The Pr(y=1|x) is plotted as a function of the covariate x. The top plot shows Category 1 functions (both RSPF and LC satisfied) which include linear logistic (black), quadratic logistic (red) and cubic logistic (green). The middle plot gives functions of Category 2 (only LC satisfied/RSPF not satisfied) which are linear scaled logistic (black), Gaussian (quadratic) (red), and cubic exponential (green) functions. The bottom plot shows Category 3 (only RSPF satisfied) which are linear logistic (black), quadratic logistic (red), and cubic logistic (green) functions

To simplify the presentation, we consider a single predictor variable x in our simulations. We use three different groups of functions for Pr(y=1|x). Category 1 meets both the RSPF and LC conditions (see the top plot of Figure 3 and top section of Table 1); Category 2 functions only satisfy the LC condition (see the middle plot of Figure 3 and middle section in Table 1) (by this we mean that the selected parametric functions achieves the probability of 1 at some points of x) and Category 3 functions only satisfy the RSPF conditions and not the LC (see the bottom plot of Figure 3 and bottom section in Table 1). In each category, three functions are selected such that it consist of one linear, one quadratic and one cubic function of x (see Figure 3 and Table 1). To ensure functions in Category 1 and Category 3 satisfy the RSPF conditions, we used exactly the same functions as those in Solymos and Lele (2015) that include linear, quadratic and cubic logistic and complementary log‐log (cloglog) functions. In Category 2, linear scaled logistic, Gaussian and cubic exponential functions are chosen as not to satisfy the RSPF conditions, because the constant terms in these functions would be cancelled out in the LK's loglikelihood function (see Equation 3). Both the scaled logistic and exponential functions were also noted in Solymos and Lele (2015) as not satisfying the RSPF conditions. These functions have been modified in our study to meet the LC conditions (see Figure 3 middle row).

**TABLE 1:** Probability of presence for the simulated species used in the experimental evaluation, where Category 1 satisfies both RSPF and LC conditions, Category 2 is where only LC is satisfied (RSPF conditions are not satisfied). Category 3 is where only RSPF conditions are satisfied (LC is not satisfied)

Category	Conditions met	Function type	Probability functions
Category 1	Local	Linear	$\mathrm{Pr}\;\left(y=1|x\right)=\frac{1}{1+\exp(‐0.606+3.64x)}$
	Certainty &	Quadratic	$\mathrm{Pr}\;\left(y=1|x\right)=\frac{1}{1+\exp(‐0.606+3.64x‐1.26x^{2})}$
	RSPF	Cubic	$\mathrm{Pr}\;\left(y=1|x\right)=\frac{1}{1+\exp(0.284+2.298x+{0.232x}^{2}‐{7.269x}^{3})}$
Category 2	Local	Scaled logistic	$\mathrm{Pr}\;\left(y=1|x\right)=\frac{8.3}{1+\exp(4‐2x)}$
	Certainty	Gaussian	$\mathrm{Pr}\;\left(y=1|x\right)=0.99\exp\left(‐{\left(4x‐2\right)}^{2}\right)$
		Exponential	Pr(y=1|x)=exp(‐0.5+0.5x $‐{0.9x}^{2}+{0.9x}^{3}$)
Category 3		Linear	$\mathrm{Pr}\;\left(y=1|x\right)=\frac{1}{1+\exp\left(‐0.5855‐1.064x\right)}$
	RSPF	Quadratic	$\mathrm{Pr}\;\left(y=1|x\right)=\frac{1}{1+\exp\left(‐0.5855‐1.064x+{0.218x}^{2}\right)}$
		Cubic	$\mathrm{Pr}\;\left(y=1|x\right)=\frac{1}{1+\exp(‐0.5855‐1.064x+0.218x^{2}+1.81x^{3})}$

**FIGURE 4:**
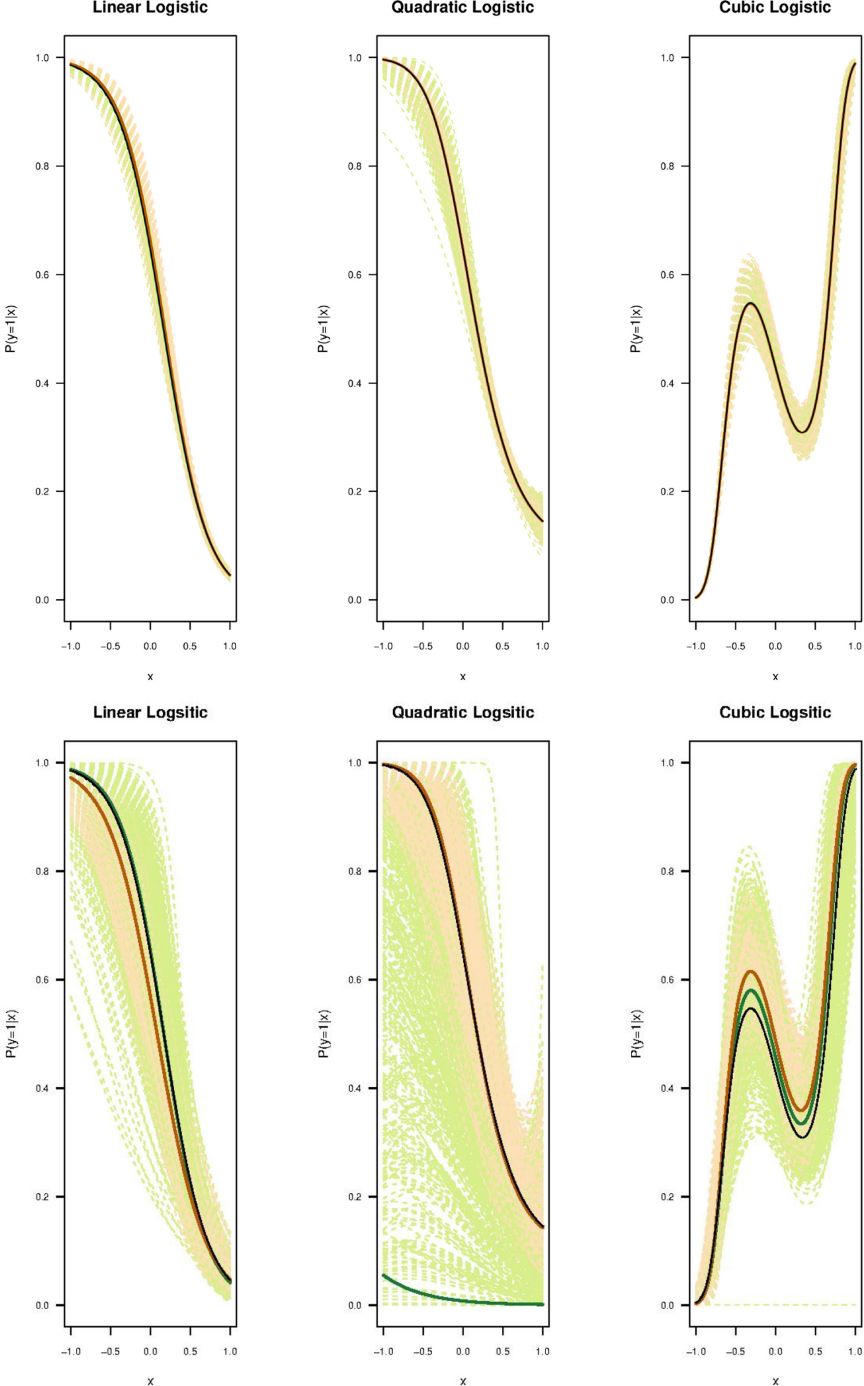
Top panel contains the graphs of Category 1, in which the Pr(y=1|x) is plotted against the covariate x when the original parametric function satisfies both the RSPF and LC conditions. For each simulation, 5000 presence samples and 50,000 background samples were chosen from a landscape with the predictor variable x in [‐1,1]. The bottom panel contains the category 1 functions with a smaller sample of 250 presence samples and 5000 background samples chosen from the same landscape as the top panel. Estimated lines from each of 1000 simulations are plotted in light green (LK) and light orange (CLK). Three solid lines are plotted representing the true probability function (black), representing the average LK estimates (green) and average CLK estimates (orange), respectively, as averaged from 1000 simulations

**FIGURE 5:**
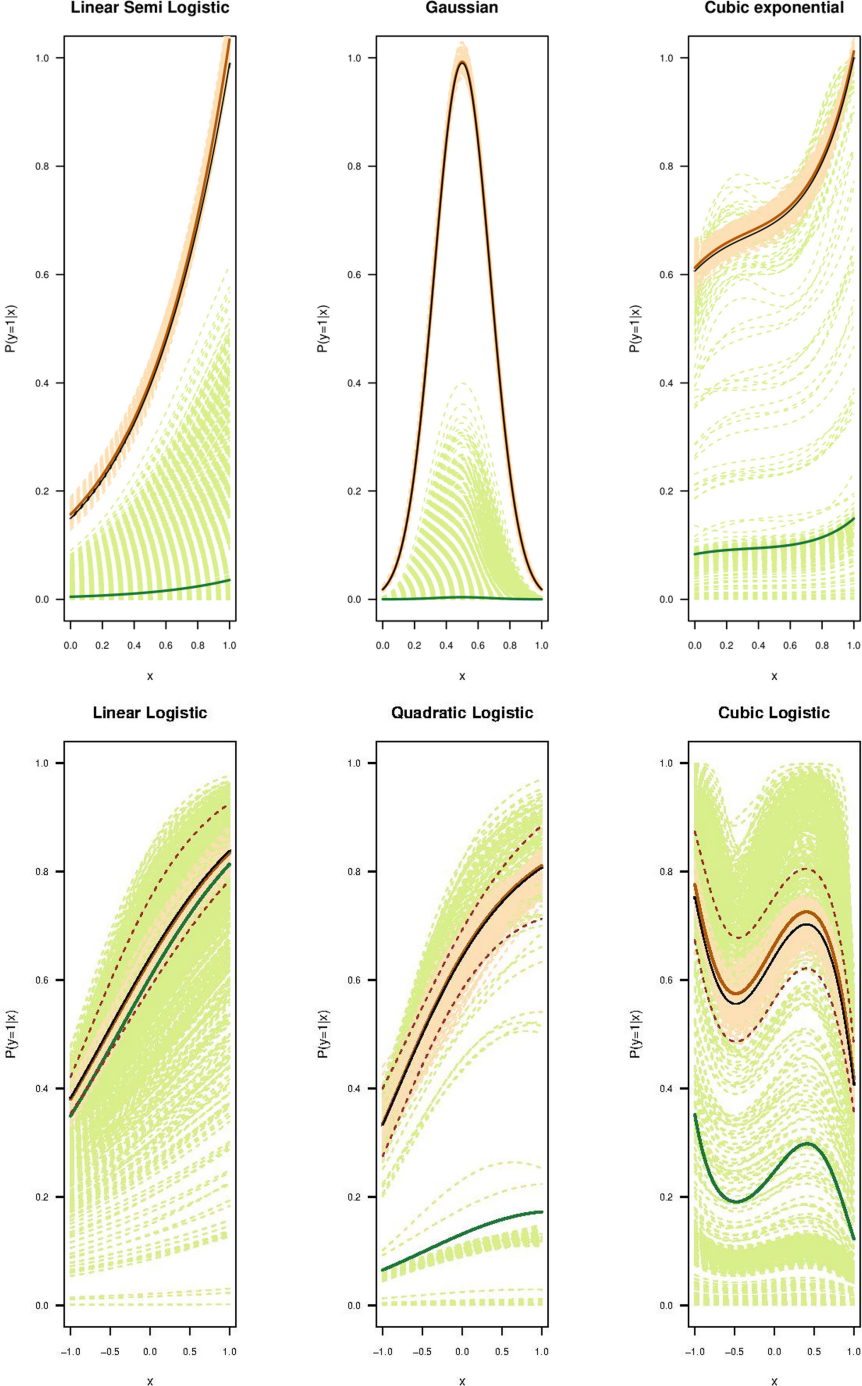
Top row shows the Category 2 results, in which only the LC is satisfied. The exponential parametric function is used to fit CLK method, while the LK methods fit by logistic function. Bottom row shows Category 3 results, where the RSPF conditions are met, with the LNK of the maximal probabilities of 0.83 used for linear logistic (left), 0.8 for Quadratic (middle) and 0.75 for Cubic (right) logistic functions respectively. Estimated lines from each of 1000 simulations are plotted in light green (LK) and light orange (CLK). Three solid lines are plotted representing the true probability function (black), average of the LK estimates (green) and average of the CLK estimates (orange), respectively, over 1000 simulations. The dashed brown lines in the bottom row show a single run of the simulation when the LKN is mis‐specified by ±10% from its true value. For each simulation, 5000 presence samples and 50,000 background samples were generated from a landscape with the predictor variable x in [0,1] and [‐1,1] for Category 2 and 3, respectively

All the functions in Categories 1 and 3 were fitted by logistic functions for both the LK and the refined CLK methods (see Figure 4 top row and Figure 5 bottom row). Gaussian and cubic exponential functions in Category 2 were fitted by exponential functions to align with the form of the original functions. The scaled logistic function in Category 2 was also fit by an exponential function to test the robustness of the CLK method with different type of fitting function than the logistic function (see Figure 5 upper row). For functions in Category 3, we assumed the LKN of Pr(y=1|x)=0.83, Pr(y=1|x)=0.8 and Pr(y=1|x)=0.75, which are the maximal probabilities of the linear, quadratic and cubic logistic functions over the range of x. We also tested the performance of the CLK method with the mis‐specified LKN information, where the LKN used differs the true probability by ±10%. Results are shown in the bottom row of Figure 5.

Solymos and Lele (2015) pointed out that if the nonlinearity on the log‐scale is weak, it may need very large sample sizes to get reasonable estimate for the LK method. To ensure a stable performance of the LK method, simulations were carried out with 5000 presence samples and 50,000 background samples. When the LK method results were not satisfactory in some experiments such as Category 3, we repeated some experiments with even larger samples of 50,000 presences and 500,000 background points to investigate the performance of LK method under very large sample conditions (Phillips & Elith, 2013; Solymos & Lele, 2015). Meanwhile the performance under the smaller sample (e.g., 250 presences and 5000 background samples) were also investigated for both methods in Category 1, and the results are given in Figure 4 bottom row.

**FIGURE D2:**
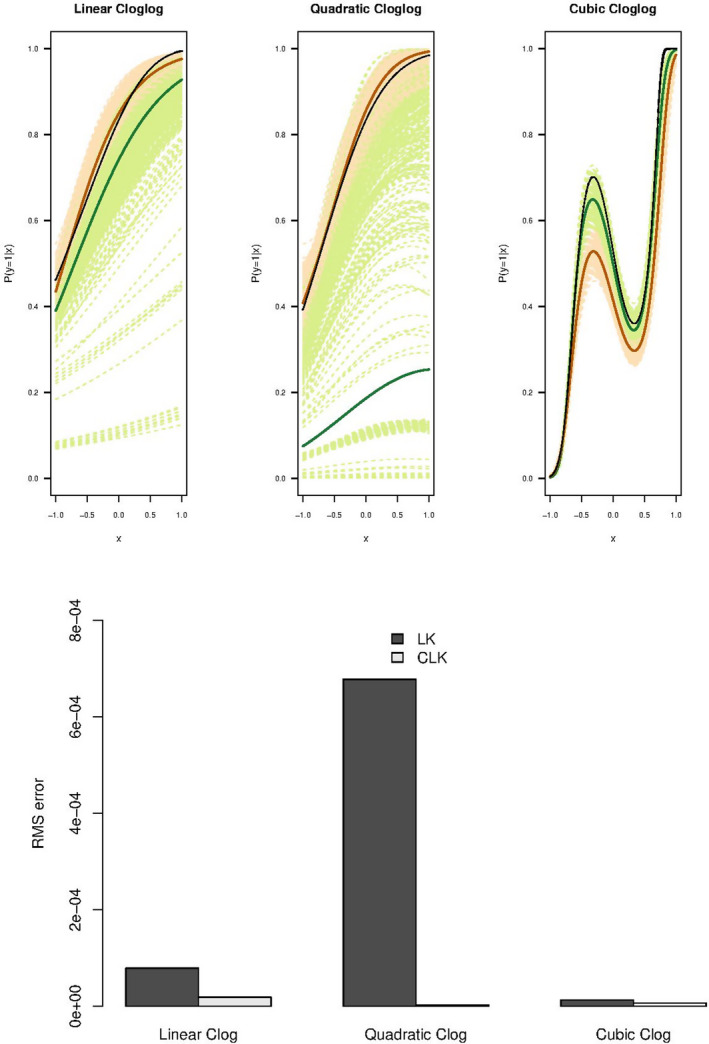
The Pr(y=1|x) is plotted when the original cloglog parametric functions that satisfy both the RSPF and the LC conditions as given by Solymos and Lele (2015). The original simulated function (black line), average LK method (green) and average CLK method (orange) are given with the respective 1000 simulation lines (light green dashed lines for LK and light orange dashed for CLK methods). For each simulation, 5000 presence samples and 50,000 background samples were chosen from a landscape with the predictor variable x in [−1, 1]. The bottom bar plot visualize the RMSEs of the two methods, LK and CLK for the above logistic functions

**FIGURE D3:**
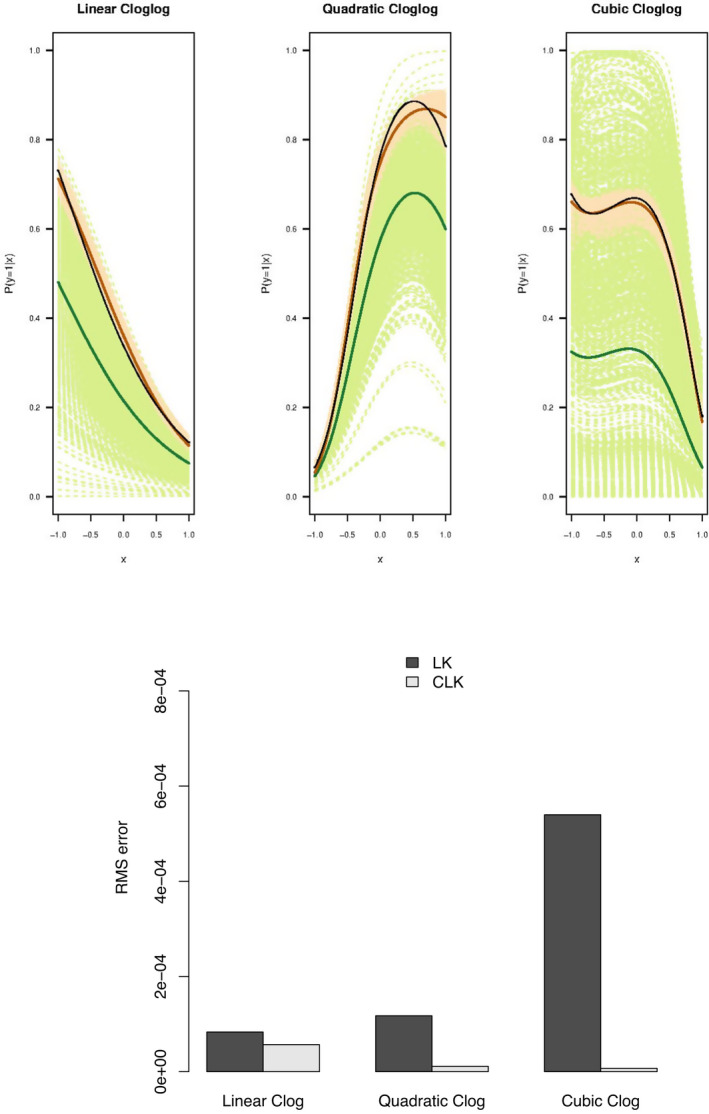
The Pr(y=1|x) is plotted when the original cloglog parametric functions that satisfy only RSPF conditions (with the LKN information assumed to be available as Pr(y=1|x)=0.73/0.89/0.68 for linear, quadratic, and cubic functions respectively at maximum point of *x*). The original simulated function (black line), average LK method (green) and average CLK method (orange) are given with the respective 1000 simulation lines (light green dashed lines for LK and light orange dashed for CLK methods). For each simulation, 5000 presence samples and 50,000 background samples were chosen from a landscape with the predictor variable x in [−1, 1]. The bottom bar plot visualize the RMSEs of the two methods, LK and CLK for the above logistic functions

The cloglog functions used in Solymos and Lele (2015) was defined as one of the possible functions satisfying the RSPF conditions. To test the robustness of both the refined CLK and the LK methods against mis‐specified parametric functional form, we generated data using cloglog function but fit the data with logistic functions. The cloglog functions studied by Solymos and Lele (2015). Results are shown in Figures D2 and D3 in Appendix D. For cloglog functions in Category 2 where only RSPF conditions are satisfied, the maximal probabilities of Pr(y=1|x)=0.73, Pr(y=1|x)=0.89 and Pr(y=1|x)=0.68 were assumed as the known LKN for fitting the linear, quadratic and cubic functions, respectively (see Figure D3).

The neural network package “nnet” in R (Venables & Ripley, 2002) was used to train Pr(s=1|x) for the refined CLK method. We rank the predicted Pr(s=1|x) for the presence and background points, and those locations whose predicted Pr(s=1|x) lie in the top 10th percentile are used as the PPL or locations with the LKN. The choice for the 10th percentile threshold is arbitrary but was tested sufficient and robust for our simulation studies.

**FIGURE 6:**
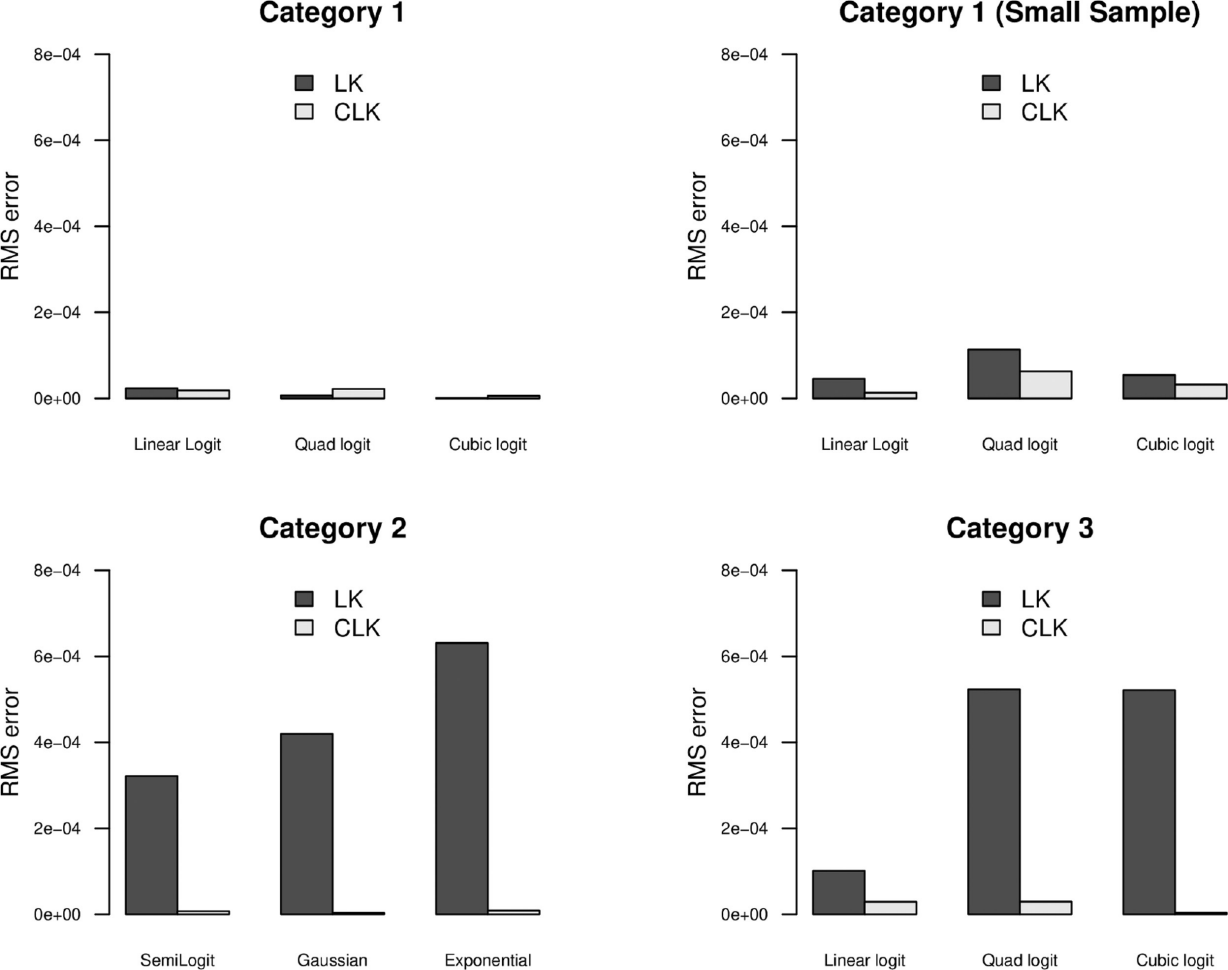
Bar plot of the RMSEs of the two methods, LK and CLK for the logistic functions in Category 1 (top left) as given in top row in Figure 4, Category 1 (top right) with small samples, Category 2 (bottom left) as given in top row in Figure 5 and Category 3 with LKN for (bottom right) as given in the bottom row of Figure 5

We run the simulations 1000 times for each species distribution function. The estimated environmental relationship for each of the 1000 simulation are plotted using the LK and refined CLK methods, against the true probabilities. Root mean square errors (RMSE) of fitted probabilities against the true probabilities of presence are also calculated and plotted in Figures 6, D2 and D3. Moreover, the error (nonconvergence) rates of the LK method in 1000 iterations are given in Tables E1 and E2 in Appendix E.

All model fitting and assessment have been carried out in R version 3.6.1. The R code supplied by Solymos and Lele (2015) has been used to choose the functions that satisfy the RSPF condition. The “ResourceSelection” package (Lele, 2009; Lele & Keim, 2006; Solymos & Lele, 2015) has been used to fit the LK method, and the R code provided by Phillips and Elith (2013) has been used with modification where relevant.

### 2.7 Revisiting an Example Hastie and Fithian (2013)

One of the pivotal publications by Hastie and Fithian (2013) demonstrated the failure of the LK method to fit and distinguish between the scaled and full logistic functions. From the perspective of ML, the presences and absences generated from a scaled logistic function are actually not separable, because scaled logistic functions do not satisfy the LC condition. We revisit the example in Hastie and Fithian (2013) and fit the scaled logistic function with the CLK method but using the LKN information. We will show that the LKN assumption is not only helpful but also necessary in removing the multiple solutions that create identifiability issues in the LK method.

**FIGURE 7:**
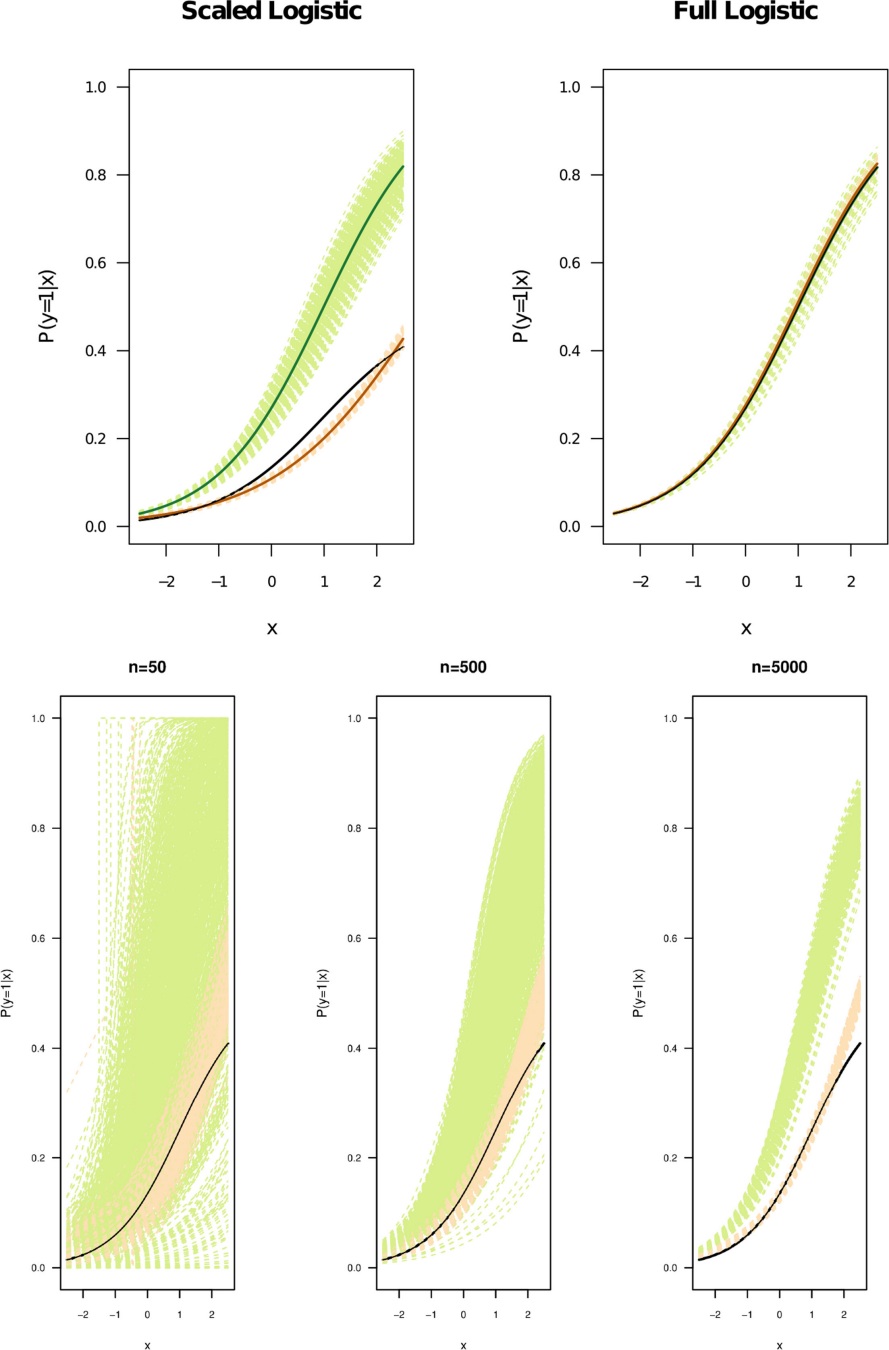
Top panel shows the full and scaled logistic functions (same as Hastie & Fithian (2013)). Here, we used the LKN of Pr(y=1|x)=0.8 for the full logistic function and Pr(y=1|x)=0.4 for the scaled logistic function both around x=2.5. The green and orange dashed lines (top panel) represent estimates of LK and CLK methods, respectively, and the solid green and orange lines represent the averages of the 1000 simulations. The original logistic functions are given in black. Bottom panel shows the fitted scaled logistic functions from both the LK method (green) and the CLK (orange) with different sample sizes. From the bottom left to right, the presence and background samples increase from (50, 5000), (500, 5000) to a very large samples of (5000, 50,000)

In their paper, Hastie and Fithian (2013) demonstrated the lack of fit in the LK methods using two logistic functions. The full logistic function considered is $f_{1}\left(x\right)=\frac{1}{1+\exp(1‐x)}$ while the scaled logistic function is exactly half of that, i.e., $f_{2}\left(x\right)=\frac{1}{2}f_{1}\left(x\right)$, where x∈[‐2.5,2.5]. We first fit both functions by the LK and CLK methods, using a sample of 5000 presences and 50,000, background data points with 1000 iterations (top row of Figure 7). We also tried different sample size to test the robustness of the LK method and the CLK method (see bottom row of Figure 7). For the refined CLK method, we assumed the LKN of Pr(y=1|x)=0.8 and Pr(y=1|x)=0.4, which are the maximal probabilities that the full and scaled logistic functions can reach over the range of x. We used the top 10th percentile of sites where Pr(s=1|x) are highest as the locations with LKN.

## 3 RESULTS

### 3.1 Simulation results

The results of the three categories are given in Figures 4 and 5 in which the Pr(y=1|x) is plotted against x. In these figures, the black line gives the original parametric functions selected as given in Table 1. The green and orange dashed lines indicate the simulation lines for the 1000 iterations carried out for the LK and refined CLK methods while the respective solid lines show the average of those simulations. The top panel of Figure 4 shows that when both the RSPF and the LC conditions are satisfied in Category 1, the LK and refined CLK methods both perform well in estimating the true function of Pr(y=1|x), with the mean estimates close to the true functions. Both methods have similarly small RMSEs for the fitted probabilities against the true probabilities (see top left panel of Figure 6). However, when the number of presence points reduces (such as to 250), the LK method starts to perform unstably with more variations (see Figure 4 bottom row). The dispersion for the CLK method also increase but are consistently stable. Overall, the CLK method with the LC condition perform well with small samples regarding both the accuracy and precision (see top right plot in Figure 6).

Category 2 functions (Figure 5 top panel) do not satisfy the RSPF conditions, but they meet the LC conditions. Under these circumstances, the refined CLK method performs consistently well for all functions (orange), while the LK (green) performs poorly as expected given that these functions fail to satisfy the RSPF conditions. The poor performance is seen by the simulated curves not close to the original functions (black) and with large deviations (light green lines) that indicate possible identifiability issues. RMSEs of the LK method are very large in contrast to the small RMSEs of the refine CLK method (Figure 6 bottom left panel).

Figure 5 bottom panel shows the performance of the two methods for functions in Category 3. Interestingly, the LK method (green lines) fails to perform well in all functions in Category 3, where the claimed RSPF conditions are all met for these functions (Solymos & Lele, 2015). The average fit for the linear logistic function may appear close to the true probability function, but the individual estimates are so unstable that fluctuate widely. A large dispersion is sometimes a signal that the model is not identifiable, and more information is required for the model to be identified. This also poses a large risk of using the LK method in practice, as it could give a result that may be far away from the actual probability of presence. On the other hand, the refined CLK method well predicts the true probability function resource selection probability function when accurate LKN information is available, with much smaller variances compared to those of the LK method. This is partially due to more information utilised. Using the neural network to train Pr(s=1|x) also improves the accuracy of our method compared to the logistic regression classification method.

The brown dashed lines in the bottom panel of Figure 5 show the estimate of probability when the LKN is mis‐specified by a ±10% margin of error from the true LKN. Overall, a 10% mis‐specification of the LKN has led to a similar level (i.e. around 10%) of over or under‐estimation of the probabilities of presence. Regardless, the biased estimates of the refined CLK method are still more accurate and precise than the LK estimates for both the quadratic and cubic logistic functions. Although the mean of LK seems a better fit for the linear logistic function (Figure 5 left bottom plot), the LK shows an exceptionally large dispersion, an indicator of the model lack of identifiability. The bottom right‐side panel of Figure 6 again shows the larger RMSEs of the LK method compared with the refined CLK method for all three functions in Category 3.

Comparable results were also observed for cloglog functions (see Appendix D), where both methods were fitted using logistic functions. In this circumstance, the refined CLK method consistently performs well with accurate estimates and small variances. Even though the cloglog function satisfies the RSPF conditions, performance of the LK method is unstable and not robust as claimed by Solymos and Lele (2015). These simulations again demonstrate that parametric function based conditions are fragile and insufficient. The LKN additional to the LK method provides a robust and solid solution to estimate the true probability of presence from PB data (see Figures D2 and D3 in Appendix D).

**FIGURE B1:**
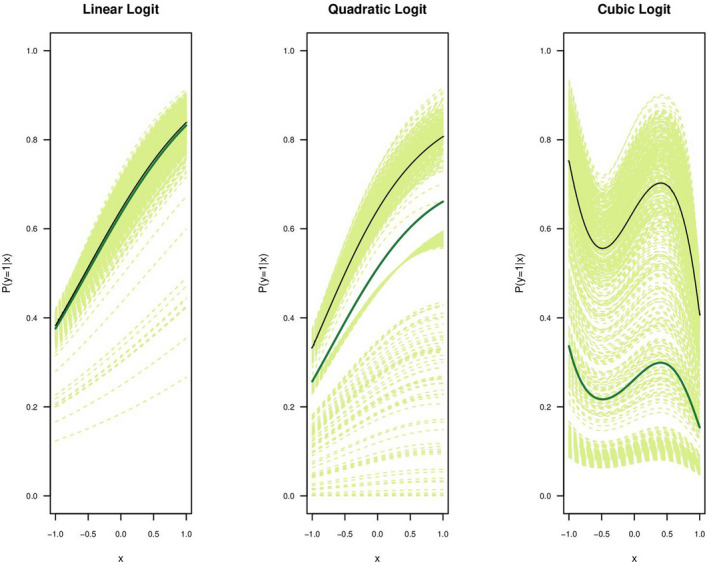
Category 3: The Pr(y=1|x) is plotted when the original logistic parametric function only satisfies the RSPF condition (Category 3) for very large sample size. The original simulated function (black line), average LK method (green) is given with the respective 1000 simulation lines (light green) for the Linear Logistic (left), Quadratic Logistic (middle) and Cubic logistic (right). For each simulation, 50,000 presence samples and 500,000 background samples were chosen from a landscape with the predictor variable x in [‐1,1]

Phillips and Elith (2013) and Solymos and Lele (2015) mention that the LK method performs better when the sample size is “large enough.” Solymos and Lele (2015) hint that the above‐mentioned problem can be overcome with larger sample sizes. We found that when using a “very large” sample of 50,000 presences and 500,000 background samples (see Figure B1 in Appendix B), the LK method shows an improved performance with less variations when compared with the performance from a sample of 5000 presences and 50,000 background samples (See Figure 5). Nevertheless, a significant improvement in performance is only observed for some functions (linear logistic function in Figure B1 in Appendix B), while the improvement is only slight for other functions.

An instability of the LK method is also observed while using the “ResourceSelection” package in fitting the LK method. The LK shows a greater variation in their estimation, when different starting values were used to optimize the log‐likelihood function. Even in the cases when the logistic parametric functions satisfy the RSPF conditions, failures (nonconvergences) were observed in our simulations. The nonconvergence rate (see Table E1 in Appendix E) is the proportion of simulations that didn't converge. Using the quadratic logistic function in Category 1 as an example, the LK method didn't converge 2.2% of times when the function satisfies the LC condition. The failure rate increases to 31.4% (for the quadratic logistic function in category 3) when the LC is not met, while in both cases, the quadratic functions still satisfy the RSPF condition. Similar behavior is also observed for mis‐specified models (See Table E2 in the Appendix E).

### 3.2 Results of the revisited example from Hastie and Fithian (2013)

Hastie and Fithian (2013) showed that the proportional likelihood (see Equation 2) that forms the basis of the LK method has identifiability issues. In their argument, they considered the full logistic function $f_{1}\left(x\right)$ and a scaled logistic $f_{2}\left(x\right)$ which is exactly half of the full logistic function $f_{1}\left(x\right)$. The top panels in Figure 7 plots these two functions $f_{1}\left(x\right)$ & $f_{2}\left(x\right)$ given by Hastie and Fithian (2013).

Because of the scaling, it is easy to see from Equation 3 that the likelihoods of these two functions $f_{1}\left(x\right)$ and $f_{2}\left(x\right)$ are exactly the same. Thus, when we attempted to fit the two different datasets (generated from the scaled and the full logistic function), the LK method gave exactly the same parameter estimates for the $\beta_{i}$ for both functions. This is confirmed in our simulation when large sample sizes are applied. The LK method yields the same estimates for both the full and the scaled logistic functions. Thus despite the fact that $f_{2}\left(x\right)=\frac{1}{2}f_{1}\left(x\right)$ (black line in Figure 7 top left panel), the estimation scheme finds ${\hat{f}}_{2}\left(x\right)\approx {\hat{f}}_{1}\left(x\right)$. Figure 7 (top left panel) shows the LK fits (green lines) are close to the full‐logistic (plotted in black Figure 7 top right panel). This effect was observed and explained in Hastie and Fithian (2013). The refined CLK method, on the other hand, predicts both logistic functions very well, when the maximal probabilities for Pr(y=1|x) are available for both the full and scaled logistic regressions. The predicted functions from the refined CLK method align closely with both the true probability functions, with small variations observed between replications (see orange dashed lines in the top panel of Figure 7).

An effect that was not discussed in Hastie and Fithian (2013) is the case when the sample sizes of the presence and/or background are small (bottom panel of Figure 7). The identifiability problems of the LK method are even more severe in these cases. The green dashed lines show that the many LK fits we attempted are in fact a manifestation of the likelihood's multiple proportional solutions interfering. Each such solution has the same likelihood as the full logistic model. As the sample sizes increase, the model gradually identifies the function with the “maximum” likelihood as the interested parametric function, which in these types of cases tends to be the full logistic function. This is why the LK method has such trouble fitting scaled functions. Moreover, we also found that it requires very large samples for the LK method to perform stably (bottom panel of Figure 7). By contrast, the CLK method performs consistently well even with a small number of presence points.

**FIGURE C1:**
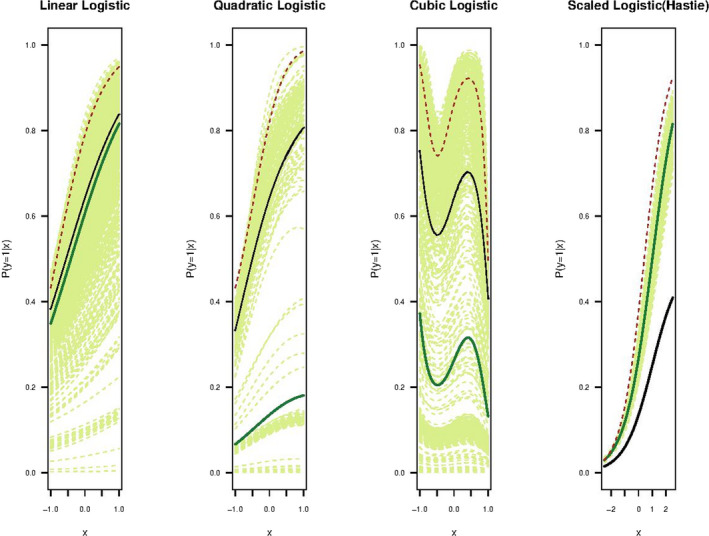
Simulations were conducted for logistic functions in Category 3 and the scaled logistic function Hastie where the LC are not met but are erroneously assumed. The original simulated function (black line), average of the refined CLK method (brown dashed line), and average of the LK method (solid green) are shown, with the respective 1000 simulated lines (light green) for the LK method. From left to right, it shows the Linear Logistic (left most), Quadratic Logistic (middle left), Cubic logistic (middle right) and scaled logistic function (right most). For each simulation, 5000 presence samples and 50,000 background samples were chosen from a landscape with the predictor variable x in [‐1,1]

Simulations in Appendix C show the performance of the refined CLK method for logistic functions in Category 3 and the scaled logistic function where the LC conditions were not satisfied but were erroneously assumed. Results show that the average probabilities (dashed red lines in Figure C1) are overestimated proportionally to the true probabilities of presence (black lines). Because of the (wrong) LC restriction, the maximal estimated probabilities tend to reach one. Compared to the LK method, however, the mean estimates of the refined CLK method are still closer to the true parametric functions apart from the linear logistic function, where the LK method again shows exceptionally large variation that indicates possible identifiability issue with the method. This issue is only alleviated when sample size becomes extremely large (see Figure B1 in Appendix B).

## 4 DISCUSSION

In this paper, we have studied the modeling of the probability of presence (or probability of use) of a species using presence‐background (or use‐availability) data. To estimate the resource selection probability, either a strong parametric assumption such as the logit function requirement for the LK method or extra information such as the population prevalence (as in the SB, EM, and CLK methods) has been proposed (Elith et al., 2011; Phillips & Elith, 2013; Wang & Stone, 2019; Ward et al., 2009).

The LK method, along with other methods with a strong parametric assumption, have been widely used in the literature and they claimed that the absolute probability of presence may possibly be estimated without requiring an estimate of the prevalence (Lele & Keim, 2006; Royle et al., 2012; Solymos & Lele, 2015). But there have been arguments in the literature and many researchers have warned against using these methods to accurately estimate the actual probability of presence (Elith et al., 2006; Guillera‐Arroita et al., 2015; Hastie & Fithian, 2013; Keating & Cherry, 2004; Knape & Korner‐Nievergelt, 2015; Phillips & Elith, 2013; Renner & Warton, 2013; Wang & Stone, 2019; Ward et al., 2009). Our simulation study suggests that the LK method performs well only when the “RSPF‐eligible” functions satisfy the LC condition and samples are not small. When presence sample is small, linear logit/cloglog functions with the LC seem the only functions that performs reasonably well. Taking a large sample along with a strong parametric assumption may sometimes overcome these problems, as we think Solymos and Lele are arguing. But either finding a large enough sample or knowing a parametric assumption is not feasible in practice. Our paper shows again that these methods (such as the LK) are fragile and can give unreliable estimates even when its underlying so‐called RSPF condition is met. Thus, our results contradict the claim made in Solymos and Lele (2015) that “if the RSPF condition is satisfied, it is possible to estimate absolute probability of selection.” Although the RSPF condition is necessary for the LK method, our study indicates the “success” of the LK method depends crucially on the LC, that is, classes of presence and absence are separable.

It was shown that the LK method has difficulty distinguishing between the actual likelihood and other possibly similar likelihoods that arise from scaled functions. From the perspective of ML, the presence observations generated from the scaled logistic functions, which do not satisfy the LC condition, completely overlap with absence observations and two classes are inseparable. It is thus expected that the LK method would have multiple solutions and difficulty in locating the true probability function of presence. Under this circumstance, we believe that not only the LK method but any other methods should have trouble in distinguishing two inseparable classes, except when extra information is enforced. The extra information, however, if coming from unfounded model assumption, only renders fragile estimation/prediction of the desired probabilities. Rather, the LKN proposed in the paper provides an effective additional datum to reliably estimate absolute (rather than relative) probability of presence from the PB data. This condition relaxes the commonly recognised population prevalence and helps overcome the identifiability issue inherent in the proportional LK likelihood function, that is, the problem Hastie and Fithian (2013) elaborated on.

Other related ideas of anchoring the presence probability also exists, such as using extra effort at a subset of sites (e.g., with occupancy model designs) or integration of presence‐only with presence‐absence data (Dorazio et al., 2015; Fithian et al., 2015; Koshkina et al., 2017). The conceptual idea behind all these methods is the same, that is, using additional information to anchor the PB data to estimate probability of presence. We propose a new approach which combines both ML (Elkan & Noto, 2008; Li et al., 2011) and statistical techniques to strengthen the estimation of population prevalence and to finally estimate the probability of presence. Our approach estimates the population prevalence based on the LC assumption. By doing this, we eliminate the strong parametric assumption in the LK method, while removing the need of additional fieldwork to estimate the population prevalence. Our estimate of the population prevalence can also be fed into the SB, EM as well as the widely used MAXENT (Phillips et al., 2006) models to predict the absolute probability of presence.

The LC condition assumes that Pr(y=1|x)=1 is satisfied at the PPL (Li et al., 2011). The LC (or the PPL) was also defined as the “positive subdomain/anchor set” assumption in the ML literature as a key assumption for the population prevalence to be identifiable in a balanced ecosystem (Bekker & Davis, 2020). As we described, users can find PPL by identifying large prediction values of Pr(s=1|x) from the observed presences and background points (or the presence locations). As one reviewer pointed out, users could enlarge the study area so that it is more likely to include certain PPL in study area, because under the LC or the positive subdomain assumption there will be locations for which Pr(y=1|x)=1 (Bekker & Davis, 2020; Elkan & Noto, 2008; Li et al., 2011). Additionally, adding more relevant features(covariates) to distinguish between species’ presences and absences may also help. In ecological system, LC holds, for example, if some locations or combination of niches always provide sustainable conditions for a species to survive. Long‐term spatial persistence and spatial stability is well known for forest communities where some species and individuals persist on centennial to millennial timescales, in coral reefs, and even in seagrass meadows where structurally complex habitats create persisting localised hotspots. These are just several examples where LC would seem to be a very reasonable assumption and will be dependent on suitability of surrounding ecological and environmental conditions.

In some cases, the LC condition may not be satisfied but was still erroneously assumed. The probability of presence at each location would be overestimated but the ranking of the presence probabilities across study area is still preserved. Under the circumstance, the LKN condition is proposed as an extension of the LC condition to overcome the identifiability issue underlying those “inseparable” presence and absence problems. There exists potential risk of mis‐specifying the LKN for attention, where a mis‐specified information will give biased probability of presence. The level of bias depends on the degree of mis‐specification.

The PBL method (Li et al., 2011) can predict the required probability well, but it is hard to gain the inferential relationship between the probability of a species’ presence and the influential environmental covariates. The CLK method, on the other hand, provides a remedy by finding the impact of the environmental covariates on species’ presence, whilst also providing good predictions. We also extend the LC or the PPL condition to the more general LKN condition. Similar to the PBL method, the performance of the refined CLK method is dependent on the classification method being chosen to fit the conditional labelling probability Pr(s=1|x). In our numerical studies, a simple logistic regression as the classifier gave poor results when compared to the neural network (comparative results not shown). That is the reason we used the neural network in classifying Pr(s=1|x) versus Pr(s=0|x). Similar as other ML methods, our proposed approach also requires reasonably large background samples to enable reliable estimation, whereas the selection of large background samples can be easily achieved in practice.

An alternative suggestion by a referee is to perform/conceive the CLK method in a Bayesian framework, given its similarity to setting a highly informative prior (i.e., a spike prior) on the LKN at some sites. This could be a potential research to extend the CLK method in the framework of Bayesian analysis. Meanwhile, this paper assumes presence sample is SCAR, where there is either no sampling bias or the sampling effort and presence of species are conditionally independent givenx. When SCAR is not satisfied and if the sampling mechanism is known, such as depending on certain covariates, the information could be incorporated into a model to enable an unbiased estimation of the probability of presence. This suggests a potential direction for future research to combine the ML and statistical methods to study PB data with sampling bias.

## 5 CONCLUSION

Estimating the absolute probability of presence from presence only data is an ill‐defined problem. In order for the probability identifiable, additional assumption is necessary. This paper first shows that the proper condition to estimate the absolute probability of presence is not the RSPF conditions as the LK method has claimed. We found that the LK method is fragile and often fails to give reliable estimates even when the RSPF conditions are satisfied. Rather, we propose the LKN condition, which relaxes the predetermined population prevalence condition that has so often been used in much of the existing literature. The proposed concept of LKN extends the LC or the PPL assumption when the latter is not satisfied. The concept has significant implications for demonstrating the necessary assumption to possibly estimate the absolute probability of presence from PB data in species distribution modelling.

## AUTHOR CONTRIBUTIONS

**Yan Wang:** Conceptualization (lead); Formal analysis (equal); Funding acquisition (lead); Methodology (lead); Supervision (supporting); Writing—original draft (equal); Writing—review and editing (lead). **Chathuri L. Samarasekara:** Conceptualization (supporting); Formal analysis (equal); Methodology (supporting); Writing—original draft (equal); Writing—review & editing (supporting). **Lewi Stone:** Conceptualization (equal); Methodology (supporting); Supervision (supporting); Writing—original draft (supporting); Writing—review and editing (supporting).

## CONFLICT OF INTEREST

There are no conflicted of interest from the authors.

## Figures and Tables

**FIGURE D1:**
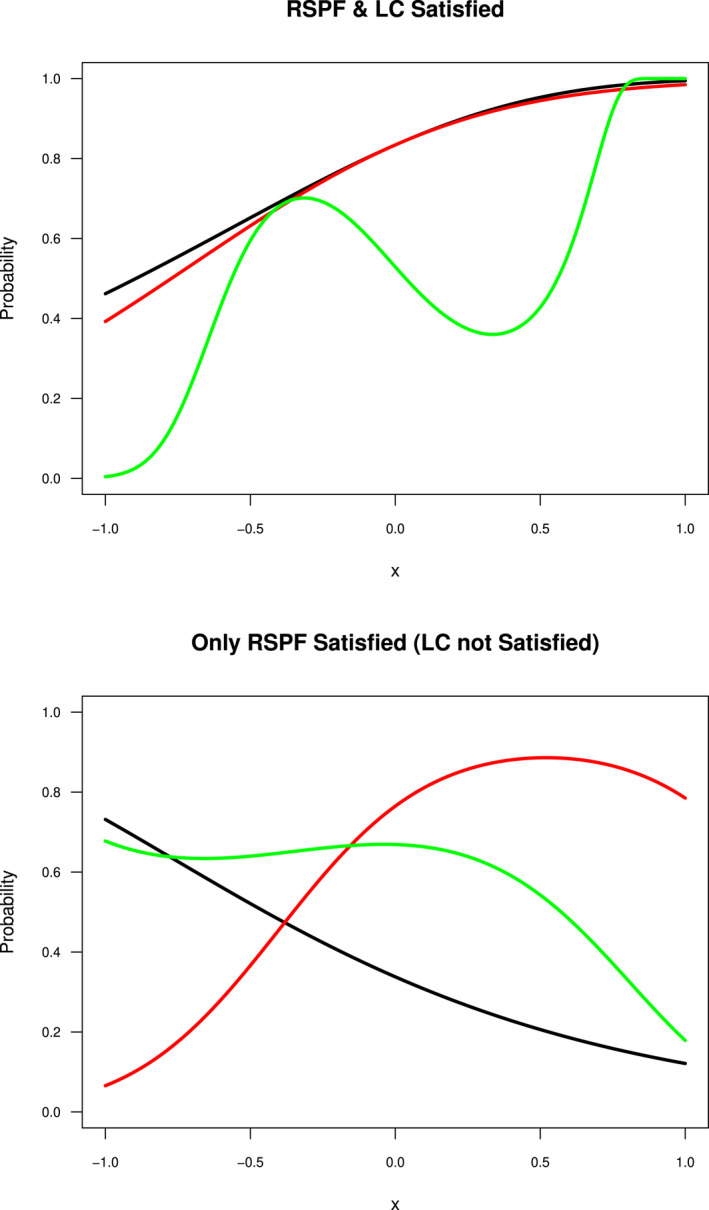
The selected simulated species distributions of the two groups of cloglog functions; category 1 and 2, respectively. The plots display the following functions named as follows. The top plot gives the functions of the category 1: only RSPF conditions satisfied, that are linear (black), quadratic (red) and cubic (green). The bottom plot of category 2: RSPF and LC satisfied gives the linear (black), quadratic (red) and cubic (green)

## Data Availability

The data that support the findings of this study are available in Github at https://github.com/chathuri‐sam/Refined_CLK.git. These data were derived from the following resources available in the public domain: Phillips and Elith (2013) with https://doi.org/10.6084/m9.figshare.c.3305961.v1, and R packages at https://github.com/psolymos/detect/tree/master/extras/revisitingSV.

## APPENDIX A

### RELATIONSHIP BETWEEN THE ABSOLUTE PROBABILITY OF PRESENCE AND THE CONDITIONAL PROBABILITY OF SELECTION

The relationship is identified using Li et al. (2011) and Phillips et al. (2009). The definition of Pr(s=1|x), that is, the probability that a site will be chosen as a presence sample rather than a background sample, conditioned on the environmental variables (Phillips et al., 2009) is not similar to Pr(y=1|x), that is, the probability of occurrence conditioned on the environmental variables. We will now define the notations used.

We denote presence as y=1 and absence as y=0. Hence, the desired model can be written as P(y=1|x). Let π=Pr(y=1), the overall prevalence of the species of presence. and $p_{1}$ is the number of observed presence data points in the presence sample in the training data set. The background data in the training set contain $p_{2}$ presences and $n_{2}$ absences, both in proportion to their overall population prevalence and $p_{2}+n_{2}=n_{0}$. Moreover, let s=1 denote the observed presence and s=0 the background data. Remember that a background data point is either an unknown presence or an unknown absence. If s=1, we know that y=1; however, if s=0, we do not know whether y=1 or y=0. Therefore,

$$\mathrm{Pr}\;\left(s=1\right)=\frac{p_{1}}{\left(p_{1}+n_{0}\right)},$$

$$\mathrm{Pr}\;\left(s=0\right)=\frac{n_{0}}{\left(p_{1}+n_{0}\right)}.$$

The background points are drawn independently and identically from all locations in the study area. It contains $p_{2}$ presences, which are in proportion to the overall population prevalence π. Therefore, $\mathrm{Pr}\;\left(y=1\right)=\pi =\frac{p_{2}}{n_{0}}$.

By definition of conditional probability, we have,

$$\mathrm{Pr}\;\left(s=1|x\right)=\frac{\mathrm{Pr}\;(x,s=1)}{\mathrm{Pr}\;(x)}$$

By applying the Bayes rule, we have

(A1)
$$\begin{array}{c} \begin{array}{cc} \text{Pr}\;(s=1|x) & =\frac{\text{Pr}\;\left(x|s=1\right)\text{Pr}\;\left(s=1\right)}{\text{Pr}\;\left(x\right)}\\ & =\frac{\text{Pr}\;\left(x|s=1\right)\text{Pr}\;\left(s=1\right)}{\text{Pr}\;\left(x|s=1\right)\text{Pr}\;\left(s=1\right)+\text{Pr}\;\left(x|s=0\right)\text{Pr}\;\left(s=0\right)}. \end{array} \end{array}$$

Then by definition of Pr(s=1) and Pr(s=0)

$$\mathrm{Pr}\;\left(s=1|x\right)=\frac{p_{1}\cdot \mathrm{Pr}\;\left(x|s=1\right)}{p_{1}\cdot \mathrm{Pr}\;\left(x|s=1\right)+n_{0}\cdot \mathrm{Pr}\;\left(x|s=0\right)}.$$

Next, dividing all terms by $p_{1}\cdot \mathrm{Pr}\;\left(x|s=1\right)$, we have

$$\mathrm{Pr}\;\left(s=1|x\right)=\frac{1}{1+\frac{n_{0}}{p_{1}}\cdot \frac{\mathrm{Pr}\;(x|s=0)}{\mathrm{Pr}\;(x|s=1)}}.$$

Pr(x|s=1) is the density of the environmental covariates conditional on the observed presence, while Pr(x|s=0) is the density of environmental covariates for the entire region. Pr(x|s=1) can be shown the same as the density of environmental covariates conditional on species’ presence as follows:

$$\begin{array}{c} \begin{array}{cc} \text{Pr}\;(x|s=1) & =\text{Pr}\;\left(x|s=1,y=1\right)\left(\text{by the sampling scheme}\right)\\ & =\frac{\text{Pr}\;(s=1|x,y=1)\text{Pr}\;(x|y=1)}{\text{Pr}\;(s=1|y=1)}\left(\text{by Bayes’ theorem}\right)\\ & =\text{Pr}\;\left(x|y=1\right)\left(\text{by assumption of “selected completely at random”}\right). \end{array} \end{array}$$

Similarly, as the background data is an independent and identical sample randomly selected from all locations in the whole region, so Pr(x|s=0)=Pr(x) the density of covariates of the whole region. These assumptions can also be found from previous work (Bekker & Davis, 2020; Lancaster & Imbens, 1996; Li et al., 2011; Phillips et al., 2009).

By applying the Bayes rule again to Pr(x|y=1) we get

$$\text{Pr}\;\left(s=1|x\right)=\frac{1}{1+\frac{n_{0}}{p_{1}}\cdot \frac{\text{Pr}\;\left(x\right)}{\text{Pr}\;(x|y=1)}}=\frac{1}{1+\frac{n_{0}}{p_{1}}\cdot \frac{\text{Pr}\;\left(x\right)}{\frac{\text{Pr}\;\left(y=1|x\right)\text{Pr}\;\left(x\right)}{\text{Pr}\;\left(y=1\right)}}},$$

Hence,

$$\text{Pr}\;\left(s=1|x\right)=\frac{1}{1+\frac{n_{0}}{p_{1}}\cdot \frac{\text{Pr}\;\left(y=1\right)}{\text{Pr}\;(y=1|x)}}=\frac{1}{1+\frac{n_{0}}{p_{1}}\cdot \frac{\pi }{\text{Pr}\;(y=1|x)}}=\frac{1}{1+\frac{p_{2}}{p_{1}}\cdot \frac{1}{\text{Pr}\;(y=1|x)}}.$$

If we define $c=\frac{p_{1}}{p_{1}+p_{2}}$, the above equation becomes

(A2)
$$\text{Pr}\;\left(s=1|x\right)=\frac{1}{1+\frac{1‐c}{c}\cdot \frac{1}{\text{Pr}\;(y=1|x)}}.$$

Equation (A2) shows the important relationship between the model fitted to PB data using a presence–absence method and the target probability of presence. It can see that the fitted probability of Pr(s=1|x) is not equal to, or even proportional to probability of presence (Elith et al., 2011; Li et al., 2011; Phillips et al., 2009).

Here, we also show the relation between c and the sampling probability of Pr(s=1|y=1) as follows:

$$\begin{array}{c} \begin{array}{c} \begin{array}{cc} \frac{\text{Pr}\;(s=1|x)}{1‐\text{Pr}\;(s=1|x)} & =\frac{\text{Pr}\;\left(x|s=1\right)\text{Pr}\;\left(s=1\right)}{\text{Pr}\;\left(x|s=0\right)\text{Pr}\;\left(s=0\right)}=\frac{\text{Pr}\;\left(x|y=1\right)\text{Pr}\;\left(s=1\right)}{\text{Pr}\;\left(x\right)\text{Pr}\;\left(s=0\right)}\\ & =\frac{\text{Pr}\;\left(y=1|x\right)\text{Pr}\;\left(x\right)}{\text{Pr}\;\left(y=1\right)\text{Pr}\;\left(x\right)}\cdot \frac{\text{Pr}\;\left(s=1\right)}{\text{Pr}\;\left(s=0\right)}\\ & =\frac{\text{Pr}\;(y=1|x)}{\text{Pr}\;\left(s=0\right)}\text{Pr}\;\left(s=1|y=1\right). \end{array} \end{array} \end{array}$$

We see from A2 that $\frac{\mathrm{Pr}\;(s=1|x)}{1‐\mathrm{Pr}\;(s=1|x)}=\frac{c}{1‐c}\mathrm{Pr}\;\left(y=1|x\right)$. Therefore, $\frac{\mathrm{Pr}\;(s=1|y=1)}{\mathrm{Pr}\;(s=0)}=\frac{c}{1‐c}$, and thus $c=\frac{\mathrm{Pr}\;(s=1|y=1)}{\mathrm{Pr}\;(s=1)+\mathrm{Pr}\;(s=1|y=1)}$.

## APPENDIX D

### RESULTS OF THE COMPLEMENTARY LOG‐LOG (CLOGLOG) FUNCTIONS

The chosen cloglog functions are given in Figure D1 and Table D1. These cloglog functions are divided into two categories, category one having three functions that satisfy both the RSPF and the LC condition and the second category only satisfies the RSPF conditions. These functions are also fitted using the logit parametric function to investigate the performance of the LK and CLK methods when the model is mis‐specified. The results given in the Figures D2 and D3 show that the performance of the CLK method is good even though the models are mis‐specified, whereas the LK method is still unable to accurately identify the original function as expected.

**TABLE D1:** Probability of presence for the simulated complementary log‐log (cloglog) species used in the experimental evaluation

Simulated species	Probability of presence
Category 1	Both RSPF & LC Satisfied
Linear	Pr(y=1|x)=1‐exp(‐exp(‐0.886‐1.16x))
Quadratic	$\mathrm{Pr}\;\left(y=1|x\right)=1‐\exp(‐\exp\left(0.37+1.56x‐1.5x^{2}\right))$
Cubic	$\mathrm{Pr}\;\left(y=1|x\right)=1‐\exp(‐\exp\left(0.5855+1.064x‐0.218x^{2}‐1.81x^{3}\right))$
Category 2	Only RSPF conditions Satisfied
Linear	Pr(y=1|x)=1‐exp(‐exp(0.5855+1.064x))
Quadratic	$\mathrm{Pr}\;\left(y=1|x\right)=1‐\exp(‐\exp\left(0.5855+1.064x‐0.218x^{2}\right))$
Cubic	$\mathrm{Pr}\;\left(y=1|x\right)=1‐\exp(‐\exp\left(0.1‐0.064x‐0.85x^{2}‐0.81x^{3}\right))$

## APPENDIX E

### NONCONVERGENCE RATE OF LK METHOD

These tables show the nonconvergence rate of the LK method when fitting the models. It is also noted that the CLK method did not have any errors/nonconvergences during model fitting.

**TABLE E1:** Proportion of nonconvergence for the LK methods fitting three categories of logit functions with 1000 replications of simulations. 5000 presence samples and 50,000 background samples were used in simulations

Category (Logistic)	Conditions satisfied	Function	Nonconvergence rate
Category 1	Local Certainty	Linear	0
	&	Quadratic	2.2
	RSPF	Cubic	0
Category 2		Gaussian	10.5
	Local Certainty	Scaled Logistic	0.3
		Exponential	38
Category 3		Linear	1.2
	RSPF	Quadratic	31.4
		Cubic	11.6

**TABLE E2:** Proportion of nonconvergence for the LK methods fitting two categories of complementary log‐log (cloglog) functions with 1000 replications. 5000 presence samples and 50,000 background samples were used in simulations

Category (cloglog)	Conditions satisfied	Function	Nonconvergence rate
Category 1	Local Certainty	Linear	3.5
	&	Quadratic	22.9
	RSPF	Cubic	0
Category 2		Linear	0.1
	RSPF	Quadratic	1.5
		Cubic	16.2
